# ATP-Binding Pocket-Targeted Suppression of Src and Syk by Luteolin Contributes to Its Anti-Inflammatory Action

**DOI:** 10.1155/2015/967053

**Published:** 2015-07-05

**Authors:** Jeong-Oog Lee, Deok Jeong, Mi-Yeon Kim, Jae Youl Cho

**Affiliations:** ^1^Department of Aerospace, Information Engineering, Bio-Inspired Aerospace Information Laboratory, Konkuk University, Seoul 143-701, Republic of Korea; ^2^Department of Genetic Engineering, Sungkyunkwan University, Suwon 440-746, Republic of Korea; ^3^School of Systems Biological Science, Soongsil University, Seoul 156-743, Republic of Korea

## Abstract

Luteolin is a flavonoid identified *as a major anti-inflammatory component* of *Artemisia asiatica*. Numerous reports have demonstrated the ability of luteolin to suppress inflammation in a variety of inflammatory conditions. However, its exact anti-inflammatory mechanism has not been fully elucidated. In the present study, the anti-inflammatory mode of action in activated macrophages of luteolin from *Artemisia asiatica* was examined by employing immunoblotting analysis, a luciferase reporter gene assay, enzyme assays, and an overexpression strategy. Luteolin dose-dependently inhibited the secretion of nitric oxide (NO) and prostaglandin E_2_ (PGE_2_) and diminished the levels of mRNA transcripts of inducible NO synthase (iNOS), tumor necrosis factor- (TNF-) *α*, and cyclooxygenase-2 (COX-2) in lipopolysaccharide- (LPS-) and pam3CSK-treated macrophage-like RAW264.7 cells without displaying cytotoxicity. Luteolin displayed potent NO-inhibitory activity and also suppressed the nuclear translocation of NF-*κ*B (p65 and p50) via blockade of Src and Syk, but not other mitogen-activated kinases. Overexpression of wild type Src and point mutants thereof, and molecular modelling studies, suggest that the ATP-binding pocket may be the luteolin-binding site in Src. These results strongly suggest that luteolin may exert its anti-inflammatory action by suppressing the NF-*κ*B signaling cascade via blockade of ATP binding in Src and Syk.

## 1. Introduction

*Artemisia asiatica* Nakai is medicinal plant used as a clinically prescribed drug for treating gastritis and gastric ulcer in Korea [1, 2]. Indeed, it was found that an ethanolic extract of this plant can suppress the formation of gastric ulcers induced by HCl/EtOH in mice [3]. Previous phytochemical studies indicated that flavonoids including eupatilin, jaceosidin, and luteolin are major anti-inflammatory components [3–6]. It was found that eupatilin is capable of suppressing aortic smooth muscle cell proliferation and migration by inhibiting the activities of PI3K, MKK3/6, and MKK4 [7]. In an* in vivo* experiment, this compound also displayed a neuroprotective activity against ischemia/reperfusion-derived neuronal damage in mice [8]. Mechanistically, eupatilin was examined for its ability to suppress LPS-induced inflammatory gene expression via inhibition of nuclear factor- (NF-) *κ*B-dependent pathways.

Many studies have been performed on the anti-inflammatory action of luteolin. It has been found that this compound is capable of suppressing the production of inflammatory mediators such as tumor necrosis factor- (TNF-) *α*, interleukin- (IL-) 8, nitric oxide (NO), reactive oxygen species (ROS), and prostaglandin E_2_ (PGE_2_) and inhibiting their corresponding genes, such as inducible NO synthase (iNOS) and cyclooxygenase-2 (COX-2), in peritoneal macrophages, RAW264.7 cells, and BV2 cells [9–11]. The two pathways inhibited by luteolin have been identified as being the NF-*κ*B pathway, mediated through inhibition of phosphatidylinositol 3-kinase (PI3K) and AKT [12]; and the activator protein- (AP-) 1 pathway, mediated through inhibition of extracellular-signal-regulated kinase 1/2 (ERK1/2), p38, and c-Jun N-terminal kinase (JNK) [13, 14].

Although many researchers have studied the immunopharmacological activities of luteolin, the targets of this compound have not been fully elucidated. In this study, therefore, we undertook to identify the molecular targets of luteolin involved in the regulation of LPS-stimulated macrophages.

## 2. Materials and Methods

### 2.1. Materials

**Figure 1:**
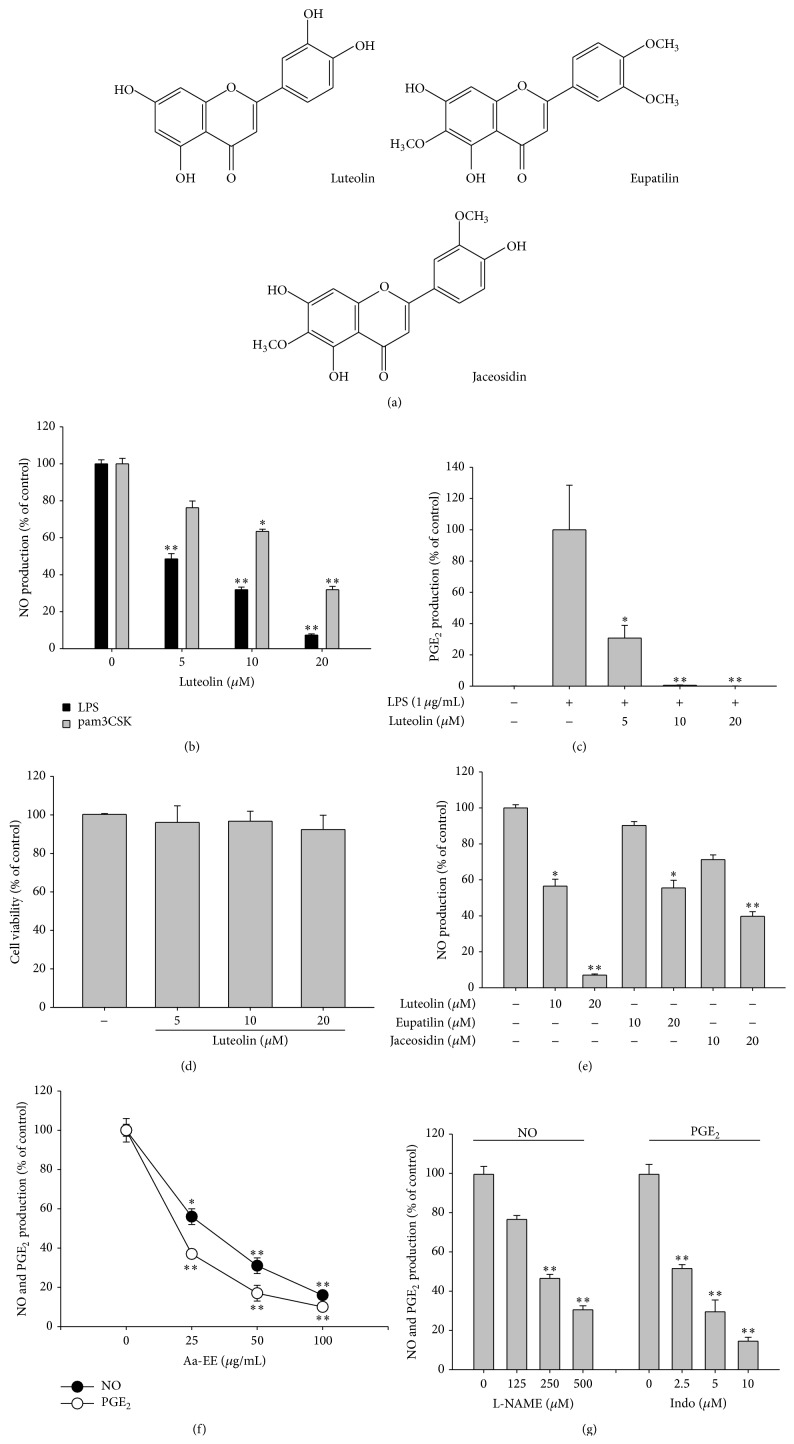
The effects of luteolin on the production of NO and PGE_2_ in macrophages, and their viability. (a) Chemical structures of luteolin, eupatilin, and jaceosidin. ((b), (c), (e), (f), and (g)) RAW264.7 cells (1 × 10^6^ cells/mL) were treated with LPS (1 *μ*g/mL) in the presence or absence of luteolin, eupatilin, jaceosidin, Aa-EE, or standard compounds [indomethacin (Indo) and L-NAME] for 24 h. The supernatants were then collected, and the NO or PGE_2_ concentrations in the supernatants were determined using the Griess assay or EIA. (d) RAW264.7 cells (1 × 10^6^ cells/mL) were treated with luteolin for 24 h. Cell viability was evaluated using the MTT assay. All data are expressed as the mean ± SD of experiments, which were performed with six samples. ^*∗*^ *p* < 0.05 and ^*∗∗*^ *p* < 0.01 compared to normal or control groups.

Flavonoids (luteolin, jaceosidin, and eupatilin) (Figure 1(a)), indomethacin (Indo), N-nitro-L-arginine-methyl ester (L-NAME), polyethylenimine (PEI), 3-(4,5-dimethylthiazol-2-yl)-2,5-diphenyltetrazolium bromide (a tetrazole) (MTT), sodium dodecyl sulfate (SDS), dimethyl sulfoxide (DMSO), pam3CSK, and lipopolysaccharide (LPS,* E. coli* 0111:B4) were purchased from Sigma-Aldrich Co. (St. Louis, MO, USA). An ethanolic extract of* Artemisia asiatica* Nakai (Aa-EE) was used as reported previously [3]. Piceatannol (*Picea*) and PP2 were obtained from Calbiochem (La Jolla, CA, USA). The enzyme immune assay (EIA) kits that were used to determine PGE_2_ levels were purchased from Amersham (Little Chalfont, Buckinghamshire, UK). Fetal bovine serum (FBS), penicillin, streptomycin, TRIzol Reagent, and RPMI1640 were purchased from GIBCO (Grand Island, NY, USA). RAW264.7 and HEK293 cells were acquired from ATCC (Rockville, MD, USA). The rest of chemical reagents used in this study were obtained from Sigma-Aldrich Co. (St. Louis, MO, USA). Phosphospecific or total-protein antibodies raised against p65, p50, c-Fos, c-Jun, inhibitor of *κ*B*α* (I*κ*B*α*), Src, spleen tyrosine kinase (Syk), p85, ERK, JNK, p38, lamin A/C, and *β*-actin were obtained from Cell Signaling Technology (Beverly, MA, USA).

### 2.2. Expression Vectors

Wild type Src (Src-WT), deletion mutants [Src kinase dead (Src-KD), Src-dSH2, and Src-dSH3] thereof, and point mutants [Src-constitutive active form (Src-CA) and Src-D404A] thereof were used as reported previously [15, 16]. Luciferase constructs containing binding sites for NF-*κ*B or AP-1 and adaptor molecules (MyD88 and TRIF) for TLR signaling were used as reported previously [17]. All constructs were confirmed by automated DNA sequencing.

### 2.3. Cell Culture and Drug Preparation

RAW264.7 cells, a murine macrophage cell line, and HEK293 cells were maintained in RPMI1640 media supplemented with 100 U/mL of penicillin, 100 *μ*g/mL of streptomycin, and 10% FBS. The cells were grown at 37°C and 5% CO_2_ in humidified air. The stock solutions of luteolin for the* in vitro* experiments were prepared in DMSO.

### 2.4. Determination of NO and PGE_2_ Production

Preincubated RAW264.7 cells or peritoneal macrophages (1 × 10^6^ cells/mL) were treated with Aa-EE (0 to 100 *μ*g/mL), its constituents [luteolin (0 to 20 *μ*M), eupatilin, jaceosidin], or standard compounds [PP2, piceatannol (*Picea*), L-NAME, or indomethacin] for 30 min. After then, LPS (1 *μ*g/mL) was further treated to the drug-treated cells for 24 h. NO and PGE_2_ production levels from 24-h culture supernatants were measured by Griess reagents and an EIA kit, as previously described [18].

### 2.5. Cell Viability Test

Before adding testing drugs to the cell suspension, RAW264.7 cells (1 × 10^6^ cells/mL) were recuperated for 18 h, and the RAW264.7 cells were incubated with luteolin (0 to 20 *μ*M) for an additional 24 h. The cytotoxic effects of luteolin were then estimated using a MTT assay, as previously reported [19, 20].

### 2.6. mRNA Analysis Using Polymerase Chain Reaction

In order to determine cytokine mRNA expression levels, total RNA was isolated from LPS-treated RAW264.7 cells using TRIzol Reagent, according to the manufacturer's instructions. Total RNA was stored at −70°C until use. Semiquantitative RT reactions were carried out as previously reported [21]. Quantification of mRNA was performed by real-time RT-PCR with SYBR Premix Ex Taq according to the manufacturer's instructions (Takara, Shiga, Japan) using a real-time thermal cycler (Bio-Rad, Hercules, CA, USA), as reported previously [22]. Semiquantitative RT-PCR was conducted as previously reported with minor modifications [23]. All of the primers (Bioneer, Daejeon, Korea) used are listed in Table 1.

### 2.7. Preparation of Cell Lysates and Nuclear Fractions for Immunoblotting Analysis

To lyse RAW264.7 or HEK293 cells (5 × 10^6^ cells/mL), these cells were added with lysis buffer prepared with cold PBS containing 1 mM sodium orthovanadate, 20 mM Tris-HCl, pH 7.4, 2 mM EDTA, 2 mM ethyleneglycotetraacetic acid, 50 mM *β*-glycerophosphate, 1 mM sodium orthovanadate, 1 mM dithiothreitol, 1% Triton X-100, 10% glycerol, 10 *μ*g/mL aprotinin, 10 *μ*g/mL pepstatin, 1 mM benzimide, and 2 mM PMSF for 30 min, with rotation, at 4°C. To remove cell debris, the lysates were centrifuged at 16,000 ×g for 10 min at 4°C and supernatants of the whole lysates were then stored at −20°C until needed. Nuclear lysates were prepared by a three-step method, as previously reported [24]. After treatment, the cells were harvested with a rubber policeman, washed with 1 × PBS, and lysed in 500 *μ*L of lysis buffer containing 50 mM KCl, 0.5% Nonidet P-40, 25 mM HEPES (pH 7.8), 1 mM phenylmethylsulfonyl fluoride, 10 *μ*g/mL leupeptin, 20 *μ*g/mL aprotinin, and 100 *μ*M 1,4-dithiothreitol (DTT) on ice for 4 min. Cell lysates were then collected using a microcentrifuge at 14,000 rpm for 1 min. During the second step, the pellet (the nuclear fraction) was washed once with wash buffer without Nonidet P-40. During the final step, the nuclei were resuspended in an extraction buffer consisting of the lysis buffer plus 500 mM KCl and 10% glycerol. The nuclei/extraction buffer mixture was frozen at −80°C and then thawed on ice and centrifuged at 14,000 rpm for 5 min. The supernatant was collected as the nuclear extract.

Whole cell or nuclear lysates were then separated by immunoblotting analysis. Proteins were loaded on 10% SDS-polyacrylamide gels and transferred by electroblotting to polyvinylidenedifluoride (PVDF) membranes. Membranes were blocked for 60 min in Tris-buffered saline containing 3% FBS, 20 mM NaF, 2 mM EDTA, and 0.2% Tween 20 at room temperature. The membranes were incubated for 60 min with specific primary antibodies at 4°C, washed 3 times with the same buffer, and incubated for an additional 60 min with HRP-conjugated secondary antibodies. The total and phosphorylated levels of p65, p50, c-Fos, c-Jun, I*κ*B*α*, Src, Syk, p85, ERK, JNK, p38, HA, lamin A/C, and *β*-actin were visualized using an ECL system (Amersham, Little Chalfont, Buckinghamshire, UK), as reported previously [25].

### 2.8. DNA Transfection and Luciferase Reporter Gene Activity Assay

Overexpression experiments were performed with HEK293 cells (1 × 10^6^ cells/mL) by transfection with Syk-WT or mutants thereof (1 *μ*g/mL each) using the PEI method in 12-well plates, as reported previously [26]. The cells were utilized for the experiments 24 h following transfection. Luteolin was added to cells 12 h before termination. For reporter gene assays, HEK293 cells (1 × 10^6^ cells/mL) were transfected with 1 *μ*g of plasmids expressing NF-*κ*B-Luc or AP-1-Luc, as well as *β*-galactosidase, using the PEI method in 12-well plates, according to the procedure outlined in a previous report [26, 27]. Luciferase Assay System (Promega, Madison, WI, USA) was used to measure luciferase activity, as previously reported [28].

### 2.9. *In Vitro* Kinase Assay with Purified Enzymes and Immunoprecipitated Enzymes

In order to determine the inhibition of the kinase activities of Src or Syk using purified enzymes, the kinase profiler service from Millipore (Billerica, MA, USA) was employed. Purified Src or Syk (human) (1–5 mU) was incubated with the reaction buffer in a final reaction volume of 25 *μ*L. After initiation of the reaction by adding Mg-ATP, the mixture was incubated for 40 min at room temperature, and then it was stopped by treatment of 5 mL of a 3% phosphoric acid solution. The reaction mixture (10 *μ*L) was then spotted onto a P30 Filtermat, and then Filtermat was washed 3 times for 5 min in 75 mM phosphoric acid and once in methanol prior to drying and scintillation counting.

In order to measure the usage of ATP by the Src kinase, Src was immunoprecipitated from lysates of WT-Src-overexpressing HEK293 cells with an anti-Src antibody. p85 was immunoprecipitated from untreated RAW264.7 cells using an anti-p85 antibody to serve as a kinase substrate. The kinase assay was carried out for 30 min at 30°C in a 50 *μ*L reaction volume including different concentrations of ATP, as well as other components, from a commercially available kinase assay kit (Upstate Biotechnology, Lake Placid, NY), as per the manufacturer's protocol and a previous report [29]. The incubation mixture was then further analyzed by immunoblotting to determine the kinase activity of immunoprecipitated Src with anti-phospho-p85.

### 2.10. Molecular Modeling Analysis

To understand the mode of binding of luteolin in the ATP-binding pockets of Src and Syk, a molecular modeling analysis of those ATP-binding pockets was performed using Sybyl version 8.02 (Tripos Associates) on the Red Hat Linux 4.0 operating system on an IBM computer (Intel Pentium 4, 2.8 GHz CPU, 1 GB memory), and the X-ray crystallographic structure of the activated Src ternary complex with the Src peptide was determined. The structure of luteolin was constructed in the Sybyl package using standard bond lengths and angles and was minimized using the conjugate gradient method. The Gasteiger-Huckel charge, with a distance-dependent dielectric function, was applied for minimization. The 106l (PDB code) structure was chosen from the Protein Data Bank, and the structure was polished using the structure preparation tool in Sybyl. Luteolin was merged at Lys-295, Met-341, and Asp-404 as it exhibits H–H bonding, and the obtained model complex was minimized using the subset minimization tool to produce a conformationally stable complex structure [30].

### 2.11. Statistical Analyses

All of the data presented in this paper are expressed as means ± SD. For statistical comparisons, results were analyzed using either ANOVA/Scheffe's* post hoc* test or the Kruskal-Wallis/Mann-Whitney test. A *p* value < 0.05 was considered to be a statistically significant difference. All statistical tests were carried out using the computer program, SPSS (SPSS Inc., Chicago, IL, USA). Similar data were also obtained using an additional independent set of* in vitro* experiments that were conducted using the same numbers of samples.

## 3. Results

### 3.1. Effect of Luteolin from* Artemisia asiatica* on Inflammatory Responses

To determine whether luteolin is able to suppress macrophage-mediated inflammatory responses, we tested the inhibitory profile of this compound using LPS- and pam3CSK-treated RAW264.7 cells. As we expected, luteolin dose-dependently blocked the production of NO in RAW264.7 cells stimulated by LPS or pam3CSK (Figure 1(b)). In addition, this compound very strongly diminished the release of PGE_2_ under the same conditions (Figure 1(c)). A cell viability test demonstrated that there was no cytotoxic activity of luteolin at its effective anti-inflammatory concentrations (Figure 1(d)). Because three major flavonoids (luteolin, jaceosidin, and eupatilin) have been identified in* Artemisia asiatica* [3, 31, 32], we next examined which flavonoid has the most potent activity. As Figure 1(e) shows, luteolin suppressed NO production to the greatest degree. We also confirmed the validity of our experimental conditions with additional experiments. Firstly, Aa-EE was able to dose-dependently inhibit NO and PGE_2_ production (Figure 1(f)), as reported previously [3]. Secondly, the standard compounds L-NAME and indomethacin (Indo) exhibited similar NO and PGE_2_ inhibitory profiles (Figure 1(g)), as reported previously [33].

### 3.2. Effect of Luteolin on Transcriptional Activation in LPS-Treated RAW264.7 Cells

**Figure 2:**
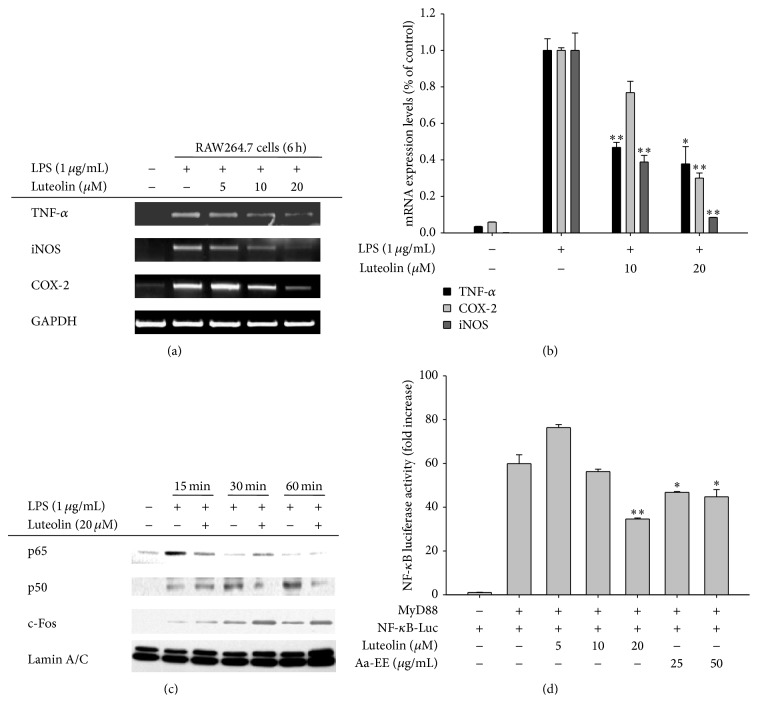
The effects of luteolin on iNOS, COX-2, and TNF-*α* gene expression and transcriptional regulation in LPS-treated RAW264.7 cells. ((a) and (b)) RAW264.7 cells (5 × 10^6^ cells/mL) were incubated with LPS (1 *μ*g/mL) in the presence or absence of luteolin for 6 h. iNOS, COX-2, and TNF-*α* mRNA levels were determined using RT-PCR (a) and real-time PCR (b). (c) RAW264.7 cells (5 × 10^6^ cells/mL) were incubated with LPS (1 *μ*g/mL) in the presence or absence of luteolin for the indicated times. After preparing nuclear fractions, the levels of total translocated transcription factors (p65, p50, c-Fos, and c-Jun) were determined by immunoblotting analysis. (d) HEK293 cells cotransfected with NF-*κ*B-Luc (1 *μ*g/mL) and *β*-gal (as a transfection control) plasmid constructs were treated with luteolin in the presence or absence of adaptor molecule (MyD88) for 12 h. Luciferase activity was determined via luminometry. All data are expressed as the mean ± SD of experiments. ^*∗*^ *p* < 0.05 and ^*∗∗*^ *p* < 0.01 compared to the control group.

To check whether the inhibitory activity of luteolin acts at the transcriptional level, we next investigated the mRNA levels of inflammation-mediating genes. As Figures 2(a) and 2(b) depict, luteolin suppressed the mRNA levels of TNF-*α*, iNOS, and COX-2 in a dose-dependent manner. Similarly, the increased nuclear levels of p50/NF-*κ*B but not of c-Fos/AP-1 or p65/NF-*κ*B by LPS were also suppressed by luteolin (Figure 2(c)). Finally, we obtained results indicating a similar inhibitory pattern via a reporter gene assay. Specifically, luteolin dose-dependently inhibited NF-*κ*B-mediated luciferase activity stimulated by MyD88, an adaptor molecule linked to TLR4 (Figure 2(d)).

### 3.3. Effect of Luteolin on Signaling Upstream of NF-*κ*B Activation

**Figure 3:**
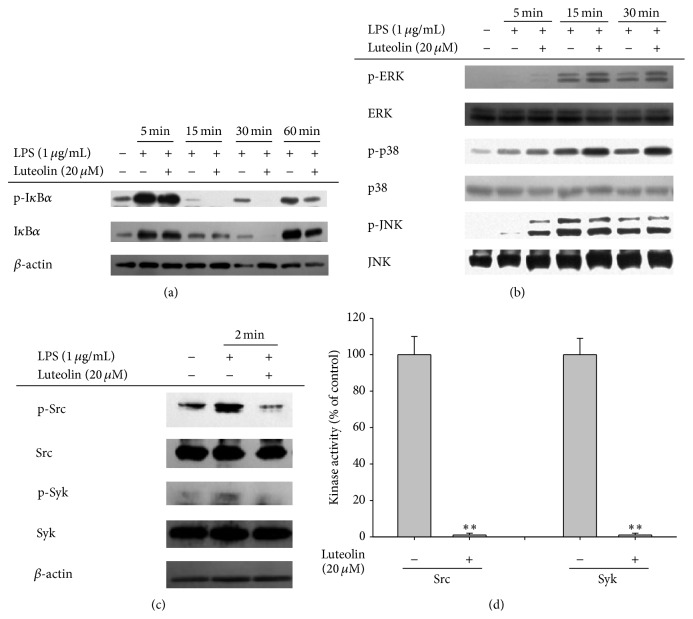
The effects of luteolin on the pathway upstream of NF-*κ*B activation. ((a), (b), and (c)) RAW264.7 cells (5 × 10^6^ cells/mL) were incubated with LPS (1 *μ*g/mL) in the presence or absence of luteolin for the indicated times. After preparing whole lysates, the levels of total or phosphorylated I*κ*B*α*, Src, Syk, ERK, p38, and JNK were determined by immunoblotting. (d) The inhibitory effects of luteolin on Src and Syk activity were determined using a conventional kinase assay with purified Src and Syk. All data are expressed as the mean ± SD of experiments. ^*∗*^ *p* < 0.05 and ^*∗∗*^ *p* < 0.01 compared to the control group.

Since we and other groups have demonstrated that inhibition of the NF-*κ*B pathway could be the chief route by which luteolin mediates its effects, the luteolin-targeted molecules in this pathway were identified through the use of immunoblotting and kinase assays. As we expected, luteolin clearly diminished phospho-I*κ*B*α* level at 15, 30, and 60 min after luteolin administration (Figure 3(a)). Unexpectedly, however, there was no inhibition of phosphorylation of ERK, p38, or JNK by luteolin, while it augmented the phosphorylation levels of these enzymes (Figure 3(b)), indicating that this compound is able to upregulate MAPK pathway rather than to inhibit. However, luteolin clearly suppressed the autophosphorylation of Src and Syk at 2 min, an optimal time to show the highest phosphorylation levels of Src and Syk, without altering the total levels thereof (Figure 3(c)). In agreement with this finding, luteolin completely suppressed the kinase activities of purified Src and Syk at a dosage of 20 *μ*M (Figure 3(d)), indicating that this compound might act as a direct inhibitor of these enzymes.

### 3.4. Mode of Inhibition of Src Kinase Activity by Luteolin

**Figure 4:**
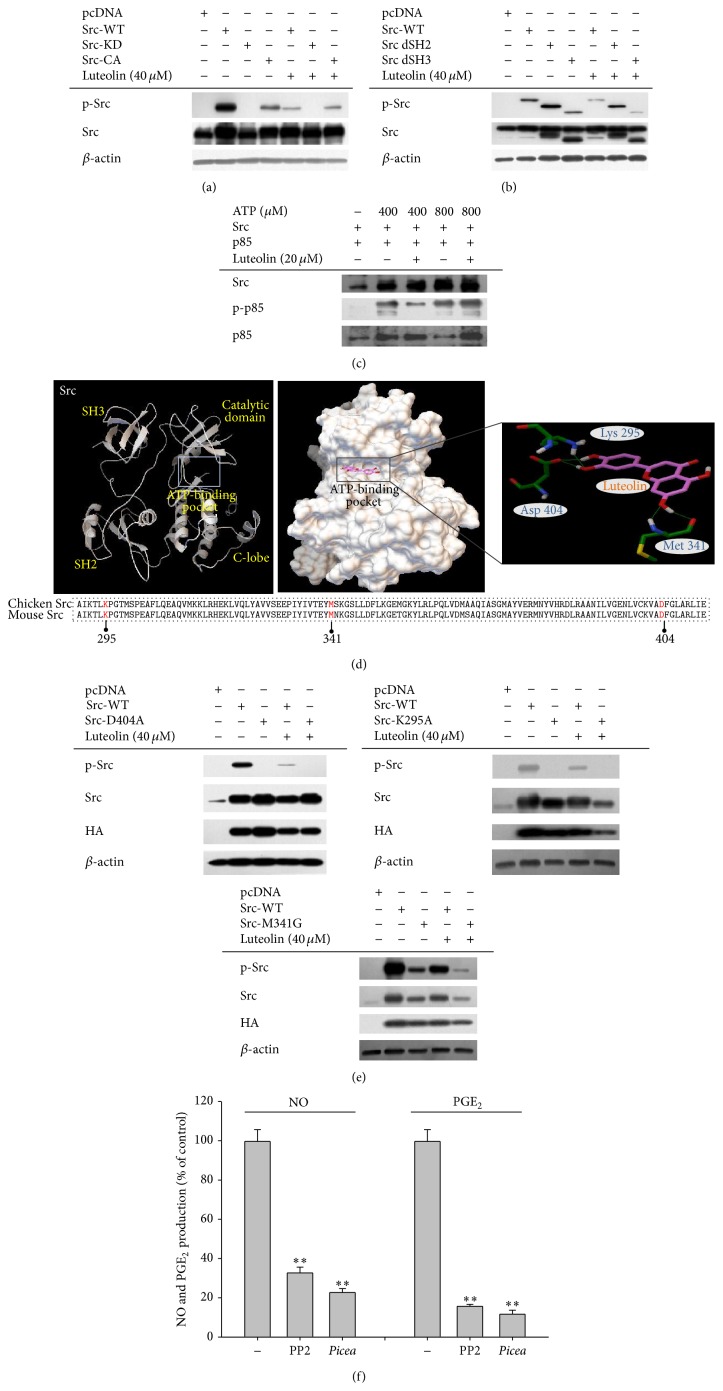
The mode of inhibition of Src kinase activity by luteolin. ((a), (b), and (e)) HEK293 cells (5 × 10^6^ cells/mL) were transfected with Src-WT, Src-KD, Src-CA, Src-dSH2, Src-dSH3, Src-D404A, Src-K295A, or Src-M341G in the presence or absence of luteolin. After preparing whole lysates, the levels of total or phosphorylated Src, HA, and *β*-actin were determined by immunoblotting. (c) Kinase assays were performed using immunoprecipitated Src as the enzyme, and immunoprecipitated p85 as the substrate, in the presence of ATP and luteolin. Src activity was determined by measuring the levels of phospho-p85 by immunoblotting analysis. (d) The putative binding site of luteolin in the ATP-binding pocket of Src. (f) The inhibitory effects of PP2 or piceatannol (*Picea*) on the production of NO or PGE_2_ were examined using the Griess assay and EIA. All data are expressed as the mean ± SD of experiments that were performed with six or three samples. ^*∗*^ *p* < 0.05 and ^*∗∗*^ *p* < 0.01 compared to the control group.

To further characterize the inhibitory mechanism of luteolin-mediated suppression of Src activity, we next examined it using both wild type and mutant Src. As Figure 4(a) shows, luteolin strongly suppressed the phosphorylation of Src-WT and Src-CA, but not nonphosphorylated Src-KD. In addition, the phosphorylation levels of deletion mutants lacking the SH2 or SH3 domains were also reduced by luteolin, indicating that the target binding sites of luteolin to Src could not be the SH2 or SH3 domains (Figure 4(b)). Since several papers have suggested that some flavonoids are able to bind to ATP-binding pockets [34], we next investigated whether ATP can compete with luteolin at the ATP-binding pocket of Src. As we expected, an increase of ATP from 400 to 800 *μ*M abrogated the suppression of Src phosphorylation by luteolin (Figure 4(c)), indicating that luteolin can block the binding of ATP at Src's ATP-binding pocket. To assume which amino acids in ATP-binding site can associate with luteolin, a docking model was constructed using previously reported structural data [35]. Thus, it is speculated that the hydroxyl groups of luteolin seemed to form hydrogen bonds with residues Lys-295, Met-341, and Asp-404, which are important conserved sites in the ATP-binding domain (Figure 4(d)). The importance of these sites was also confirmed by point mutations at these residues. In fact, these mutants (Src-D404A, Src-K294A, and Src-M341G) were not or less autophosphorylated, according to transfection work in HEP293 cells (Figure 4(e)). In addition, PP2 and piceatannol (*Picea*) (inhibitors of Src and Syk) inhibited the production of NO and PGE_2_ by LPS-treated RAW264.7 cells (Figure 4(f)).

## 4. Discussion

Although luteolin has been previously reported to exert anti-inflammatory activity via inhibition of both NF-*κ*B and AP-1 [10, 36], in the present study, we focused on understanding the molecular mechanisms underlying NF-*κ*B pathway inhibitory activity. Thus, this compound did not significantly suppress the AP-1 pathway, as evidenced by the finding that luteolin did not strongly suppress the phosphorylation of ERK, JNK, or p38 between 5 and 60 min after luteolin administration (Figure 3(b)). c-Fos, an AP-1 component, was also not decreased in the nuclear fraction of LPS-treated RAW264.7 cells but was enhanced instead (Figure 2(c)), in agreement with upregulation of ERK and p38 phosphorylation (Figure 3(b)). These results seem to raise a possibility that luteolin can negatively modulate dephosphorylation process of ERK and p38 by MAP kinase phosphatase-1. In addition, since other AP-1 subunits, such as ATF-2, FRA-1, and c-Jun, have been reported to act positively in LPS-induced inflammatory responses, the possibility exists that luteolin is able to control those factors. Nonetheless, our results seem to strongly suggest that NF-*κ*B may be the major inhibitory pathway in the immunopharmacological actions of luteolin. Accordingly, nuclear levels of p65 at 15 min, and of p50 at 30 and 60 min, were reduced by luteolin (Figure 2(c)). NF-*κ*B-mediated luciferase activity induced by MyD88 cotransfection was also significantly suppressed by 20 *μ*M of luteolin (Figure 2(d)). In addition, this compound clearly diminished the level of phospho-I*κ*B*α* at 15, 30, and 60 min (Figure 3(a)).

Previously, we determined that Src and Syk are major NF-*κ*B regulatory protein tyrosine kinases. It was found that the active forms of these proteins appear in response to LPS stimulation at early time points, between 1 and 5 min [21]. We and other groups have also demonstrated that specific inhibitors of these enzymes, namely, PP2 and piceatannol, suppress the production of NO and PGE_2_ (Figure 4(f)) and the expression of proinflammatory cytokines [37]. It has been reported that phosphorylation of Syk and Src contributes to the induction of early (within 1 h) phosphorylation of I*κ*B*α*, linked to early nuclear translocation of NF-*κ*B [21, 38], suggesting that the initial activation of NF-*κ*B might be controlled by these two kinases. In fact, suppression of Syk and Src by piceatannol and PP2 was found to inhibit early I*κ*B*α* phosphorylation and subsequent activation of NF-*κ*B [21]. The fact that luteolin suppressed nuclear translocation of p65 at 15 min and p50 at 30 and 60 min (Figure 2(c)) could be due to direct blockade of Syk and Src. Similarly, NF-*κ*B-targeting anti-inflammatory methanolic or ethanolic extracts from known medicinal plants such as* Evodia lepta*,* Rhodomyrtus tomentosa*,* Hopea odorata*, and* Cerbera manghas* and synthetic or naturally occurring compounds such as quercetin and (5-hydroxy-4-oxo-4H-pyran-2-yl)methyl 6-hydroxynaphthalene-2-carboxylate were identified as potent inhibitors of these enzymes [39, 40]. These results strongly indicate that Src and Syk could be important players in inflammatory responses and that targeting these enzymes with those modulators could contribute to their anti-inflammatory responses. We also verified that luteolin, with its strong anti-inflammatory activity, is able to directly block the kinase activity of both Src and Syk (Figure 3(d)), according to our kinase assay results. Our additional studies have also strongly supported the conclusion that there is direct binding of luteolin at the ATP-binding pockets of Src and Syk. For example, increased levels of ATP abrogated the suppressive activity of luteolin (Figure 4(c)). Furthermore, mutations at putative binding sites in the ATP-binding pocket, such as Asp-404, Lys-295, and Met-341, which are conserved between chicken and mouse, clearly diminished the phosphorylation of Src (Figures 4(d) and 4(e)), implying that these sites are critical for ATP binding and thus that luteolin may bind at these important amino acid residues, thereby mediating its suppression of these kinases. So far, however, it is not yet clear which function groups in luteolin are critical in the suppression of Src or Syk. Therefore, in future studies we will further examine this topic by exploring structure-activity relationships of structurally similar flavonoids.

**Figure 5:**
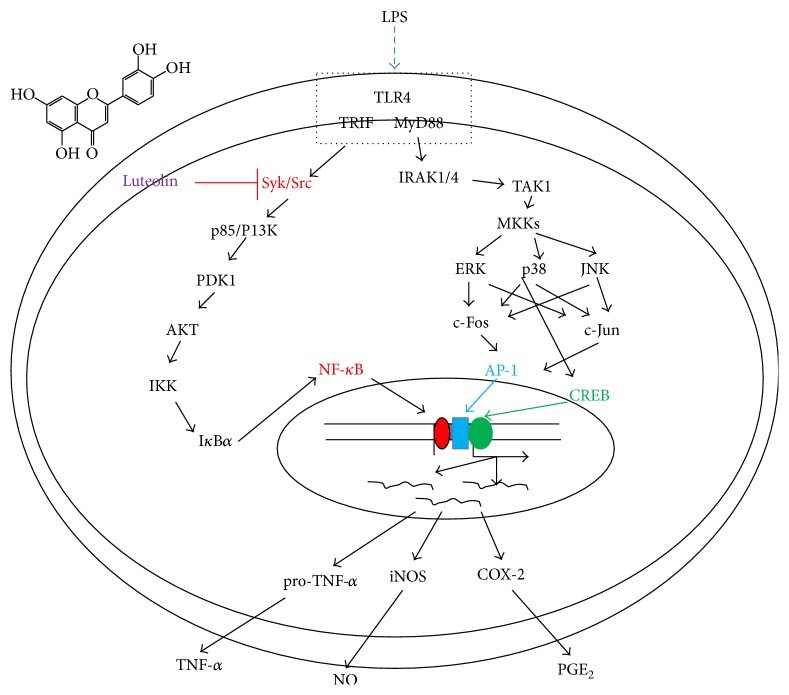
Putative mechanism of inhibition of macrophage-mediated inflammatory signaling events by luteolin.

In summary, we have shown that luteolin is capable of effectively suppressing* in vitro* inflammatory responses, in addition to possessing a radical-scavenging activity. In particular, we found that luteolin serves as a direct inhibitor of Src and Syk, thereby playing a central role in the activation of NF-*κ*B, as summarized in Figure 5. Since luteolin is present in many edible plants and fruits, we propose that luteolin-rich fractions from edible sources could be utilized for the development of therapeutic foods with anti-inflammatory properties.

## Figures and Tables

**Table 1:** Real-time PCR primers used in this study.

Name		Sequence (5′ to 3′)
iNOS	F	GGAGCCTTTAGACCTCAACAGA
	R	TGAACGAGGAGGGTGGTG

TNF-*α*	F	TGCCTATGTCTCAGCCTCTTC
	R	GAGGCCATTTGGGAACTTCT

COX-2	F	GGGAGTCTGGAACATTGTGAA
	R	GCACATTGTAAGTAGGTGGACTGT

GAPDH	F	CAATGAATACGGCTACAGCAAC
	R	AGGGAGATGCTCAGTGTTGG

## Abbreviations

PG: Prostaglandin
NO: Nitric oxide
COX: Cyclooxygenase
iNOS: Inducible NO synthase
TNF-*α*: Tumor necrosis factor-*α*
ERK: Extracellular signal-related kinase
TLR: Toll-like receptors
MAPK: Mitogen-activated protein kinase
NF-*κ*B: Nuclear factor-*κ*B
AP-1: Activator protein-1
JNK: c-Jun N-terminal kinase
EIA: Enzyme immunoassay
ELISA: Enzyme-linked immunosorbent assay
MTT: 3-(4,5-Dimethylthiazol-2-yl)-2,5-diphenyltetrazolium bromide (a tetrazole)
IRF-3: Interferon regulatory factor-3
DTT: Dithiothreitol
PI3K: Phosphoinositide 3-kinase
LPS: Lipopolysaccharide
RT-PCR: Reverse transcriptase-polymerase chain reaction.
